# Supplementary material for: Obsessive-Compulsive Disorder with Psychotic Features: Is It a Clinical Entity?
Source: Healthcare (Basel). 2022 Sep 29;10(10):1910. doi: 10.3390/healthcare10101910 (PMC9601831; doi:10.3390/healthcare10101910)
Supplement: Supplementary file 1 [file healthcare-10-01910-s001.zip › healthcare-1881177-supplementary.pdf]

healthcare

MDPI

Table S1-1. Comparison between OCD with schizophrenia (*n* =26) and OCD without psychotic disorder (*n* = 58): sociodemographic profiles and clinical characteristics.

	**OCD with schizophrenia (*n*=26) (%)**	**OCD without PD (*n*=58) (%)**	***P***
Gender			
Men	19 (73.1)	26 (44.8)	0.016
Women	7 (26.9)	32 (55.2)	
Age, mean (SD)	30.0 (10.7)	31.2 (11.9)	0.663
Marital status			
Married	0 (0.0)	14 (24.1)	0.004
Unmarried	26 (100.0)	44 (75.9)	
Housemates			
Living alone	3 (11.5)	11 (19.0)	0.533
Cohabiting or living in an institution	23 (88.5)	47 (81.0)	
Educational level			
High school or higher, including current students	18 (69.2)	51 (87.9)	0.039
Junior high school, including current students	8 (30.8)	7 (12.1)	
Present employment status			
Employed, housewife, or current student	4 (15.4)	18 (31.0)	0.182
Unemployed or on a leave of absence from duty or school	22 (84.6)	40 (69.0)	
Physical comorbidity			
Yes	9 (34.6)	18 (31.0)	0.658
No	16 (61.5)	40 (69.0)	
Family history of psychiatric illness			
Yes	16 (61.5)	29 (50.0)	0.327
No	10 (35.7)	29 (50.0)	
Psychiatric comorbidity			
Mood disorder	12 (46.2)	27 (46.5)	0.973
Anxiety disorder	10 (38.5)	16 (27.6)	0.322
Autism spectrum disorder	3 (11.5)	14 (24.1)	0.246
Self-harm attempt			
Yes	11 (42.3)	18 (31.0)	0.315
No	15 (57.7)	40 (69.0)	

The *t*-test or chi-square test was used to compare the groups, and Fisher's exact test was used if there were cells with expected frequencies of five or less.

Table S1-2 Comparison between OCD with schizophrenia (*n* =26) and OCD without psychotic disorder (*n* = 58): clinical features and measures.

**Variables**	**OCD with schizophrenia (n=26)**	**OCD without PD (n=58)**	***P***
Insight into obsessive compulsive symptoms, *n* (%)			
Poor to absent/delusional	21 (80.8)	18 (31.0)	<0.001
Good to fair	5 (19.2)	40 (69.0)	
Age at OCD onset, mean (SD)	16.1 (6.0)	19.9 (9.4)	0.028
Duration of untreated OCD (yr), mean (SD)	7.4 (7.7)	5.6 (7.4)	0.314
Duration of OCD (yr), mean (SD)	14.5 (10.0)	11.3 (9.9)	0.177

First consultation with a health care provider for OCD			
Voluntary	7 (26.9)	35 (60.3)	0.005
Involuntary	19 (73.1)	23 (39.7)	
GRID-HAMD, mean (SD)	15.9 (8.0)	16.8 (8.9)	0.684
Y-BOCS, mean (SD)	27.4 (10.0)	26.2 (9.0)	0.572
Types of obsession, *n* (%)			
Aggression	10 (38.5)	24 (41.4)	0.801
Contamination	21 (80.7)	39 (67.2)	0.297
Sexual	1 (3.8)	2 (3.4)	1.000
Hoarding	2 (7.7)	11 (19.0)	0.327
Religious	1 (3.8)	1 (1.7)	0.526
Symmetry/exactness	4 (15.4)	16 (27.6)	0.277
Somatic	3 (11.5)	11 (19.0)	0.533
Miscellaneous	8 (30.8)	21 (36.2)	0.628
Types of compulsion, *n* (%)			
Cleaning/washing	20 (76.9)	38 (65.5)	0.296
Checking	10 (38.5)	34 (58.6)	0.087
Repeating	8 (30.8)	24 (41.4)	0.355
Counting	1 (3.8)	5 (8.6)	0.661
Ordering/arranging	2 (7.7)	6 (10.3)	1.000
Hoarding/collecting	2 (7.7)	7 (12.1)	0.714
Miscellaneous	5 (19.2)	8 (13.8)	0.529
GAF, mean (SD)	23.3(13.9)	38.0 (15.9)	<0.001
CGI-S, mean (SD)	6.7 (0.47)	6.1 (1.1)	0.001

GAF = Global Assessment of Functioning; CGI-S = Clinical Global Impressions of Severity scale; GRID-HAMD = GRID Hamilton Rating Scale for Depression; Y-BOCS = Yale-Brown Obsessive-Compulsive Scale. The *t*-test or chi-square test was used to compare the groups, and Fisher's exact test was used if there were cells with expected frequencies of five or less.

.

Table S1-3. Multivariate logistic regression analysis of factors of OCD with schizophrenia (*n* =26) and OCD without psychotic disorder (*n* = 58): sociodemographic profiles, clinical features, and measures.

Variables	Level	Odds ratio	95%CI	*P*
Insight into obsessive compulsive symptoms	Poor to absent/delusional vs good to fair	0.082	0.016-0.414	0.002
Gender	Male vs female	0.391	0.104-1.467	0.164
GAF		0.934	0.880-0.992	0.027
CGI-S		1.054	0.291-3.813	0.936
Age at OCD onset		0.963	0.875-1.061	0.447
First consultation for OCD	Voluntary vs involuntary	0.640	0.133-3.071	0.577
Marital status	Unmarried vs married	0.000	0.000	0.998

CI = confidence interval.

Table S2-1. Comparison between OCD with the onset of OCD preceding the onset of psychotic disorder (*n* =24) and OCD without psychotic disorder (*n* = 58): sociodemographic profiles and clinical characteristics

	**OCD with the onset of OCD preceding the onset of PD (*n*=24) (%)**	**OCD without PD (*n*=58) (%)**	***P***
Gender			
Men	17 (70.8)	26 (44.8)	0.032
Women	7 (29.2)	32 (55.2)	
Age, mean (SD)	29.4 (11.0)	31.2 (11.9)	0.522
Marital status			
Married	0 (0.0)	14 (24.1)	0.008
Unmarried	24 (100.0)	44 (75.9)	
Housemates			
Living alone	2 (8.3)	11 (19.0)	0.327
Cohabiting or living in an institution	22 (91.7)	47 (81.0)	
Educational level			
High school or higher, including current students	16 (66.7)	51 (87.9)	0.023
Junior high school, including current students	8 (33.3)	7 (12.1)	
Present employment status			
Employed, housewife, or current student	5 (20.8)	18 (31.0)	0.426
Unemployed or on a leave of absence from duty or school	19 (79.2)	40 (69.0)	
Physical comorbidity			
Yes	9 (37.5)	18 (31.0)	0.486
No	14 (58.3)	40 (69.0)	
Family history of psychiatric illness			
Yes	15 (62.5)	29 (50.0)	0.302
No	9 (37.5)	29 (50.0)	
Psychiatric comorbidity			
Mood disorder	13 (54.2)	27 (46.5)	0.530
Anxiety disorder	9 (37.5)	16 (27.6)	0.375
Autism spectrum disorder	2 (8.3)	14 (24.1)	0.131
Self-harm attempt			
Yes	10 (41.7)	18 (31.0)	0.281
No	14 (58.3)	40 (69.0)	

The *t*-test or chi-square test was used to compare the groups, and Fisher’s exact test was used if there were cells with expected frequencies of five or less.

Table S2-2. Comparison between OCD with the onset of OCD preceding the onset of psychotic disorder (*n* =24) and OCD without psychotic disorder (*n* = 58): clinical features and measures.

Variables	OCD with the onset of OCD preceding the onset of PD (n=24)	OCD without PD (n=58)	*P*
Insight into obsessive compulsive symptoms, *n* (%)			
Poor to absent/delusional	19 (79.2)	18 (31.0)	<0.001
Good to fair	5 (20.8)	40 (69.0)	
Age at OCD onset, mean (SD)	15.3 (5.8)	19.9 (9.4)	0.008
Duration of untreated OCD (yr), mean (SD)	7.6 (7.3)	5.6 (7.4)	0.269
Duration of OCD (yr), mean (SD)	14.7 (9.9)	11.3 (9.9)	0.156
First consultation with a health care provider for OCD			
Voluntary	8 (33.3)	35 (60.3)	0.026
Involuntary	16 (66.7)	23 (39.7)	
GRID-HAMD, mean (SD)	16.9 (8.2)	16.8 (8.9)	0.941
Y-BOCS, mean (SD)	27.9 (9.6)	26.2 (9.0)	0.437
Types of obsession, *n* (%)			
Aggression	8 (33.3)	24 (41.4)	0.497
Contamination	19 (79.2)	39 (67.2)	0.424
Sexual	1 (4.2)	2 (3.4)	1.000
Hoarding	2 (8.3)	11 (19.0)	0.327
Religious	1 (4.2)	1 (1.7)	0.502
Symmetry/exactness	4 (16.7)	16 (27.6)	0.295
Somatic	4 (16.7)	11 (19.0)	1.000
Miscellaneous	8 (33.3)	21 (36.2)	0.711
Types of compulsion, *n* (%)			
Cleaning/washing	19 (79.2)	38 (65.5)	0.295
Checking	10 (41.7)	34 (58.6)	0.161
Repeating	9 (37.5)	24 (41.4)	0.744
Counting	1 (4.2)	5 (8.6)	0.666
Ordering/arranging	2 (8.3)	6 (10.3)	1.000
Hoarding/collecting	2 (8.3)	7 (12.1)	1.000
Miscellaneous	5 (20.8)	8 (13.8)	0.510
GAF, mean (SD)	22.3 (13.6)	38.0 (15.9)	<0.001
CGI-S, mean (SD)	6.7 (0.46)	6.1 (1.1)	0.001

GAF = Global Assessment of Functioning; CGI-S = Clinical Global Impressions of Severity scale; GRID-HAMD = GRID Hamilton Rating Scale for Depression; Y-BOCS = Yale-Brown Obsessive-Compulsive Scale. The *t*-test or chi-square test was used to compare the groups, and Fisher's exact test was used if there were cells with expected frequencies of five or less.

Table S2-3. Multivariate logistic regression analysis of factors of OCD with the onset of OCD precede the onset of psychotic disorder (*n* =24) and OCD without psychotic disorder (*n* = 58): sociodemographic profiles, clinical features, and measures.

**Variables**	**Level**	**Odds ratio**	**95%CI**	***P***
Insight into obsessive compulsive symptoms	Poor to absent/delusional vs good to fair	0.077	0.015-0.398	0.002
Gender	Male vs female	0.422	0.107-1.668	0.219
GAF		0.926	0.870-0.985	0.015
CGI-S		0.941	0.249-3.552	0.929
Age at OCD onset		0.960	0.870-1.058	0.409
First consultation for OCD	Voluntary vs involuntary	0.453	0.089-2.297	0.339
Marital status	Unmarried vs married	0.000	0.000	0.998
Educational level	High school or higher vs junior high school	0.478	0.096-2.386	0.341

CI = confidence interval.
